# Diagnosis of Nipple Discharge: Value of Magnetic Resonance Imaging and Ultrasonography in Comparison with Ductoscopy

**DOI:** 10.4274/balkanmedj.2016.0184

**Published:** 2017-03-28

**Authors:** Ravza Yılmaz, Ömer Bender, Fatma Çelik Yabul, Menduh Dursun, Mehtap Tunacı, Gülden Acunas

**Affiliations:** 1 Department of Radiology, İstanbul University İstanbul School of Medicine, İstanbul, Turkey; 2 Clinic of General Surgery, Okmeydanı Training and Research Hospital, İstanbul, Turkey; 3 Department of Radiology, Bakırkoy Dr. Sadi Konuk Training and Research Hospital, İstanbul, Turkey

**Keywords:** Nipple discharge, ultrasonography, magnetic resonance imaging, ductoscopy

## Abstract

**Background:**

Pathologic nipple discharge, which is a common reason for referral to the breast imaging service, refers to spontaneous or bloody nipple discharge that arises from a single duct. The most common cause of nipple discharge is benign breast lesions, such as solitary intraductal papilloma and papillomatosis. Nevertheless, in rare cases, a malignant cause of nipple discharge can be found.

**Aims:**

To study the diagnostic value of ultrasonography, magnetic resonance imaging, and ductoscopy in patients with pathologic nipple discharge, compare their efficacy, and investigate the importance of magnetic resonance imaging in the diagnosis of intraductal pathologies.

**Study Design:**

Diagnostic accuracy study.

**Methods:**

Fifty patients with pathologic nipple discharge were evaluated by ultrasonography and magnetic resonance imaging. Of these, 44 ductoscopic investigations were made. The patients were classified according to magnetic resonance imaging, ultrasonography, and ductoscopy findings. A total of 25 patients, whose findings were reported as intraductal masses, underwent surgery oincluding endoscopic excision for two endoscopic excision. Findings were compared with the pathology results that were accepted as the gold standard in the description of the aetiology of nipple discharge. In addition, magnetic resonance imaging, ultrasonography and ductoscopy findings were analysed comparatively in patients who had no surgery.

**Results:**

Intraductal masses were reported in 26 patients, 20 of whom operated and established accurate diagnosis of 18 patients on magnetic resonance imaging. According to the ultrasonography, intraductal masses were identified in 22 patients, 17 of whom underwent surgery. Ultrasonography established accurate diagnoses in 15 patients. Intraductal mass was identified in 22 patients and ductoscopy established accurate diagnoses based on histopathologic results in 16 patients. The sensitivities of methods were 75% in ultrasonography, 90% in magnetic resonance imaging, and 94.6% in ductoscopy. The specificities were 66.7% in ultrasonography, 66.7% in magnetic resonance imaging, and 40% in ductoscopy. Intraductal papillomas were mostly observed as oval nodules with well-circumscribed smooth margins within dilated ducts and persistant in the dynamic analysis. Lesions that protruded into the lumen of the ducts, either solitary or multiple, were characteristic ductoscopy findings of our patients who were diagnosed as having papilloma/papillomatosis.

**Conclusion:**

Magnetic resonance imaging and ductoscopy had no statistical superiority over each other, however they were superior to ultrasonography in the diagnosis of pathologic nipple discharge. Magnetic resonance imaging may be highly sensitive for diagnosing nipple discharge with new techniques and sequences and a non-invasive method that more advantageous for showing ductal tree visualization and is able to detect completely obstructed intraductal lesions.

Pathologic nipple discharge (PND) is characterized by spontaneous secretions that stem from unilateral single or several ducts. Among the different colours of secretions, a clear or bloody discharge is of major significance due to its higher association with breast masses (1,2,3,4). The most common cause of nipple discharge is benign breast lesions, such as solitary intraductal (ID) papilloma and papillomatosis (5). A rare but major cause of PND is breast cancer, which comprises 5%-21% of such cases (5,6,7,8). The results of cytologic examinations may be inconclusive, which leads to misdiagnosis (9).

Imaging methods used in the diagnostic approach to PND are galactography, mammography and ultrasonography (US). Magnetic resonance imaging (MRI) is a valuable adjunctive means of detecting and diagnosing ID papillomas and malignancies, especially in cases when the other three modalities are normal. In recent years, MRI has increased in importance in patients with suspicious nipple discharge or in high-risk patients with nipple discharge and it has been recommended in many studies (5,10,11,12,13). In order to increase the sensitivity and specificity of MRI in patients with PND, new techniques and different sequences from routine of breast MRI have recently been reported in several studies (14,15,16,17). With the exception of sequences and parameters used in routine breast MRI, we performed heavily T2-weighted (T2-W) fat-suppressed spin-echo sequences and other sequences in sagittal planes for the purpose of showing ductal tree visualization in patients with PND.

Ductoscopy is the imaging of mammary ducts in patients with PND using an endoscopy device, with which the inner duct can be visualized, and possible epithelial anomalies and lesions can be evaluated and their locations determined. Separately, by virtue of the functional channel of the endoscope, ID polypoid lesions can be endoscopically excised (18,19). The significance of ductoscopy has increased consistently over time.

Our aims in this study were to identify and compare the diagnostic values of US, MRI, and ductoscopy in patients with PND, and to evaluate the clinical value of MRI in the diagnosis of ID pathologies.

## MATERIALS AND METHODS

### Patients

Over a two-year period, 50 patients who presented to the outpatient department for breast diseases with intermittent or persistent pathologic nipple discharge were examined using MRI and US. After imaging evaluation, 44 patients went through a ductoscopy process, which was performed by surgeons in the surgery department. Based on the findings obtained at MRI, US or ductoscopy examinations that were reported as ID masses, 25 patients who underwent surgery including endoscopic excision for two endoscopic excision were included for diagnostic performance evaluation. The pathological results were accepted as the gold standard in the description of the aetiology of nipple discharge, and MRI, US and ductoscopy findings were compared with the pathology results of the patients who underwent surgery. In addition, MRI, US and ductoscopy findings were analysed comparatively in patients who had no surgery. Nipple aspiration fluid cytology was evaluated in all patients. This study was approved by the ethics committee. All patients gave written informed consent about the procedures to be performed.

### Ultrasonography

US examinations (Acuson Antares, Siemens, Germany) were performed on all the patients using a high-resolution linear probe with 10-12 MHz frequency within the same week after MRI. The examination was performed for both breasts in a radial and antiradial pattern, from the periphery towards the nipple. By angling the probe in the retroareolar region, the breast tissue and the duct therein were examined in detail. In 50 patients, the sonographic findings were classified as the presence of ductal dilatation, the presence of ID mass, and the presence of other lesions.

### Magnetic resonance imaging

MRI examinations were performed on a 1.5-T system with a dedicated four-channel phased-array bilateral breast coil (Achieva, Philips Medical Systems, Best, the Netherlands). All patients were examined in the prone position with their arms elevated above the head. MR protocols include a sagittal 3-dimensional (3D) heavily T2-W fat-suppressed spin-echo sequence to better visualize the ductus (TR/TE, 7000/287; matrix, 256x256; slice thickness, 2 mm; gap, 0.8; FOV, 25). The axial-sagittal precontrast fat-suppressed T1-weighted spin-echo sequences were taken (TR/TE, 350/10; matrix, 512x512; slice thickness, 3 mm; FOV, 34). In the T1-W turbo field echo (TFE) 3D sequence, dynamic sagittal images were taken once before and six times after the administration of the contrast agent at 0.2 mmol/L per kilogram of body weight. Substraction series were obtained and the time-signal intensity curves of the lesions were drawn and interpreted. In 50 patients, the MRI findings were classified as the presence of ductal dilatation, presence of ID mass, contrast involvement pattern of ID mass, dynamic contrast analysis, and the presence of other lesions. Four types of masses were identified through the evaluation of T1-W, T2-W, and subtraction images as single nodular, multiple nodular, linear, and both linear and nodular forms. We evaluated linear and nodular forms together as segmental pathology.

### Ductoscopy

Ductoscopy was performed after the patients received local anaesthesia; the procedure is not fully comfortable for patients without analgesia. In ductoscopy, a 3000 pixel LaDuScope-S optic system with 0.55 mm diameter and a 6000 pixel LaDuScope-T flex (Polydiagnost GmBH, Pfaffenhofen, Germany) operative-channelled optic system with 1.1 mm diameter were used. Ductal lavage accompanied ductoscopy during the course of the procedure. The ductoscopy results were classified according to normal findings and the presence of ID pathology. Two patients had ID polypoid lesions and subsequently underwent endoscopic excisions using microbaskets (380 μm) oriented from the operative channel of the endoscope.

### Statistical analysis

Statistical analyses were performed with SPSS 13.0 (SPSS Inc., Chicago IL, USA). Fisher’s exact test was used for the statistical analysis. A p-value less than 0.05 was regarded as statistically significant.

## RESULTS

Fifty patients were classified according to the type of discharge, its cytology, MRI and US findings, and ductoscopy. According to the MRI and US results or ductoscopy findings that were reported as ID masses, 25 patients underwent surgery and were compared with the pathology results. Additionally, MRI, US and ductoscopy findings were analysed comparatively in patients who had no surgery. The mean age of the patients was 43.3 years (range, 17-63 years) (49 female, 1 male).

### Frequency of nipple discharge, cytology results and ductal dilatation

In our study, the chief presentation of ID masses among the histopathologic patients was bloody nipple discharge. Table 1 shows the types of nipple discharge of the 50 patients. The cytology reports of two patients included atypical cells. Pathology results indicated carcinoma *in situ* and papilloma in these cases. Ductal dilatation was seen in 38 of the 50 patients, MRI and US were evaluated as 100% compatible in determining ductal dilatation.

### Comparison of MRI and histopathologic results

MRI was performed in 50 patients. According to the MRI results, ID masses were reported in 26 patients, 20 of whom underwent surgery. According to the histopathologic results, MRI established an accurate diagnosis in 18 patients. The comparison of MRI and histopathologic results in the diagnosis of ID masses is given in Table 2.

**Figure 1:**
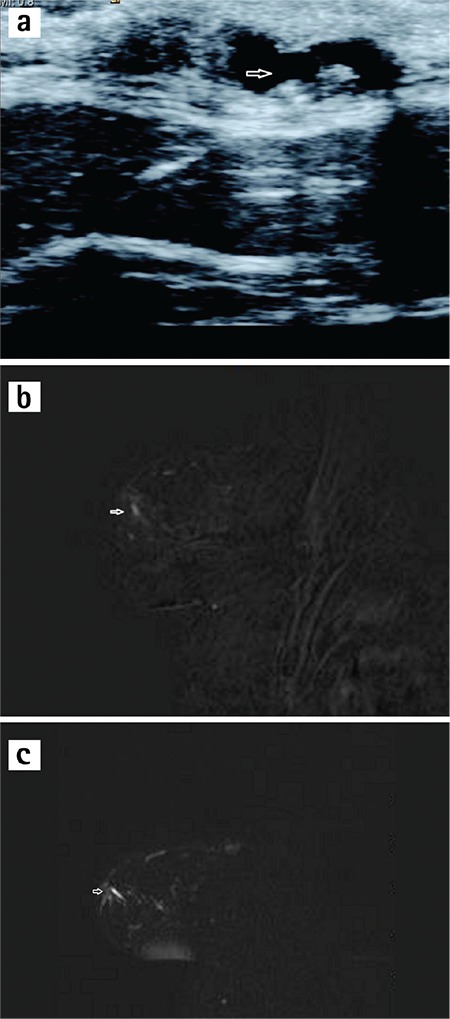
a-c. A woman aged 47 years who presented with bloody nipple discharge. She was diagnosed with papilloma as a result of an operation. Ultrasound image (a) shows multiple well-circumscribed hyperechoic nodules containing millimetric calcific components (arrow) within dilated ducts 5 mm in width. Corresponding axial T1-weighted gadolinium-enhanced subtraction magnetic resonance (MR) image (b) shows non-mass linear contrast-enhancement conforming to trace dilated duct (arrow). In sagittal heavily T2-weighted MR image (c), tubular filling defects in the duct (arrow).

**Figure 2:**
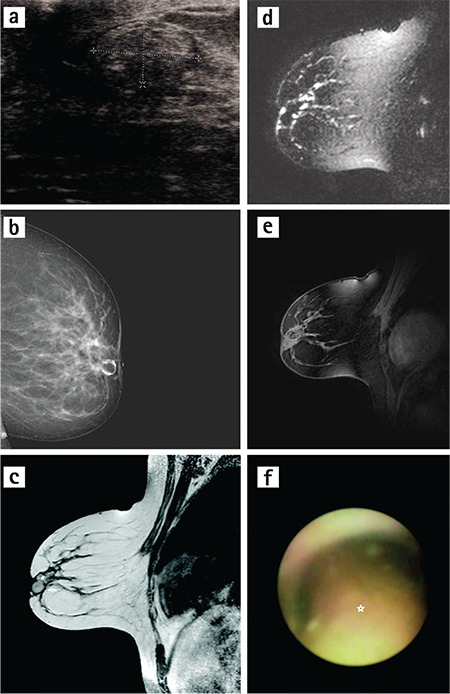
a-f. A woman aged 55 years with left serosanguineous nipple discharge. Histopathology of nodule showed intraductal papilloma and debris. Ultrasound (a) image shows heterogeneous hypoechoic intraductal nodule. Galactogram image (b) shows well-marginated filling defect, which focally expands into the retroareolar duct. Sagittal (c) precontrast T1-weighted images show spontaneous hyperintense intraductal nodule (arrow) and proteinaceous or haemorrhagic content (asterix) filled into the upper quadrant of continuing duct. In sagittal heavily T2-weighted MR image (d), nodular and tubular filling defects in the duct. Sagittal late post-contrast (e) image shows non-enhanced nodular lesions. Mammary ductoscopy (f) image shows papilloma (asterix) filled in the lumen of the duct.

In our study, MR images of ID papilloma were seen as single nodular (n=3), linear (n=6), and with segmental contrast involvement (n=2) in 11 patients whose MRI and histopathologic results were compatible (Figure 1). All lesions were hypointense on T1-W and had a variety of signal intensities on T2-W with well-circumscribed borders. In the dynamic analysis of 9 of the 11 patients, type 1 pattern was observed, whereas type 2 pattern was seen in the other cases. The pathology of the patient with the dynamic analysis result of type 3 pattern was reported as ductal carcinoma in situ; this was the only malignant result of our study. The lesion contours were irregular and a single-nodular contrast pattern was observed, as such the lesion was evaluated in MRI as suspicious for malignancy. MR images of ID papillomatosis (n=6) contrast enhancement were observed as linear (n=2) and multiple nodular (n=3). However, one patient had spontaneous hyperintense multiple nodular lesions on T1-W and no contrast on subtraction images (Figure 2). In the dynamic analysis, five lesions were evaluated as having type 1 pattern.

MRI in the diagnosis of ID mass lesions was determined to have a sensitivity of 90%, a specificity of 66.7%, the positive predictive value (PPV) was 90% and the negative predictive value (NPV) was 66.7%. The pathology results of two patients who were identified as having ID mass lesions in MRI proved to be ductal ectasia, debris and fibrosis. MRI and histopathological results were positive in 18 patients and both negative in 4 patients. In 2 patients MRI was negative but result was considered positive by pathologic evaluation.

Although both US and ductoscopy identified the pathology in one of these patients as debris and ductal ectasia, in the other case, contrary to pathology, all modalities suggested ID mass.

### Comparison of US and histopathologic results

US was performed in 50 patients. According to the US results, ID masses were identified in 22 patients, 17 of whom underwent surgery. US established accurate diagnoses based on histopathologic results in 15 patients. In our study, well-defined solid nodule(s) in a dilated duct, with/without debris were characteristic findings of ID papilloma/papillomatosis on ultrasound. For US, the sensitivity and specifity were determined as 75% and 66.7%, and the PPV and NPV were 88.2% and 44.4%, respectively.

### Comparison of ductoscopy and histopathologic results

**Figure 3:**
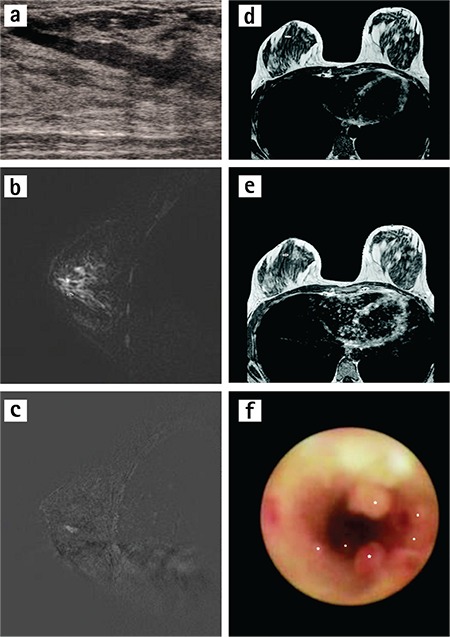
a-f. A woman aged 38 years who had had bloody discharge three times from her right breast. Pathology showed intraductal papillomatosis. Ultrasound image shows (a) shows hypoechoic masses indistinguishable from the intensive content extending along the duct. Sagittal heavily T2-weighted image (b) image shows filling defect (arrow) in distal to the duct. Sagittal T1-weighted subtraction (c) image shows non-mass linear contrast enhancement during the segment of 12 mm trace to duct. Axial T1-weighted precontrast (d) and post-contrast (e) images show contrast enhancement (arrows). After irrigation and washing; mammary ductoscopy (f) image shows papillomatosis (asterix) in the duct.

Ductoscopy was performed in 44 patients. According to the ductoscopy results, USG and histopathological results were positive in 15 patients and both negative in 4 patients. In 2 patients ultrasonography was negative but result was considered positive by pathologic evaluation. USG finding was positive but histopathological result was negative in 2 patients. ID mass was identified in 22 patients and ductal ectasia-debris in 7. Ductoscopy established accurate diagnoses in 16 patients. Lesions that protruded into the lumen of the ducts, either solitary or multiple, were characteristic ductoscopy findings of our patients who were diagnosed as having papilloma/papillomatosis (Figure 3). In the ductoscopy of 15 patients, ducts were evaluated as being normal, and similarly to the MRI and US results of these patients, there were no findings except dilatation, for which follow-up was recommended. No ductoscopy was performed on three patients whose MRI and US results identified ID lesions; instead, they were directly referred for surgery at their own request. In addition, three patients had no ID pathology in MRI and US with remission in their nipple discharge, and as such they did not undergo ductoscopy but were recommended for follow-up alone. All patients were regarded as stable because they had no or very little discharge at the 6-month follow-up examination, no additional examinations were performed.

The sensitivity of ductoscopy in the diagnosis of ID mass lesion was determined as 94.6%, and its specifity was 40%, whereas PPV was ascertained as 84.2%, and the NPV was 66.7%. The pathology results suggested ductal ectasia, debris and fibrosis in three patients who were diagnosed as having ID mass lesions in ductoscopy. Ductoscopy and histopathological results were positive in 16 patients and both negative in 2 patients. In one patient ductoscopy was negative but result was considered positive by pathologic evaluation. Ductoscopy finding was positive but histopathological result was negative in 3 patients. A summary of the performance of the various diagnostic techniques for detecting ID anomalies is shown in Table 3.

## DISCUSSION

In the evaluation of PND, the limitations of mammography and galactography have led to further research on complementary methods such as MRI and ductoscopy. This study compared the sensitivity and specificity of US, MRI and ductoscopy in cases of PND and evaluated the importance of MRI in the diagnosis of ID pathologies.

In this study, the US sensitivity in breast imaging was found higher in the identification of ID lesions than that in the literature (20,21). We revealed sensitivity and specificity for US as 75% and 66.7%, respectively. Ohlinger et al. (22) reported a sensitivity of 82.9%, higher than our study, but they calculated a specificity of 17.9%, which was lower than many studies. Ohlinger et al. (22) study was multicentred, so it is difficult to standardize diagnostic criteria; this high rate of sensitivity could in part be due to broad positive findings (e.g. ductal ectasia and cystic lesions). Our study showed that US sensitivity and specificity can be higher if used specifically for ID pathologies and PND.

Studies conducted in recent years have highlighted the importance of MRI in patients with PND whose disease is not diagnosed with used routinely imaging methods. It is also considered that the MRI, which has such a high sensitivity in the identification of cancer, would have a valuable place owing to its high sensitivity in determining ID lesions and evaluating nipple discharge (5,13,14,15,23). The term MR ductography [indirect MR galactography (MRG)] is derived from special imaging techniques such as the 3D heavily T2-W fat-suppressed sequence. The indirect MRG (idMRG) can also display the distal part of the obstructed ductus, which cannot be visualized using galactography. In a study conducted by Hirose et al. (14), contrasted 3D images and 3D heavily T2-W images were combined within a single image, thanks to which the relationship between the ID lesion and the ductus was more readily evaluated. In order to visualize the ID lesions better in MRI, Schwab et al. (15) and Wenkel et al. (16) used a different method. In direct MRG (dMRG), T1-W volumetric interpolated breath-hold examination and T1-W fast low-angle shot 3D were used after the injection of a contrast agent diluted with saline into the discharging ductus. In Schwab’s study, dMRG was reported to be superior to idMRG in the identification of ID lesions. Wenkel et al. (16) compared direct MRG with conventional galactography and finally reported that direct MRG may serve as an alternative method for preoperative duct visualization in patients who are not candidates for X-ray mammography (16). Also, Kurian et al. (17) used this technique in a high-risk patient with cytologic atypia and reported that it was a feasible and well-tolerated procedure for women at high-inherited risk of breast cancer.

Ductoscopy has an ever-increasing importance in clarifying the origin of ID proliferation and nipple discharge. It is especially advantageous for detecting partially obstructive lesions. Therefore, studies have compared ductoscopy with standard diagnosis methods (20,21,22,24). In a study conducted by Grunwald et al. (20), ductoscopy was compared with mammography, galactography, US, MRI, cytology and pathology. It was reported that the utrasound had the highest sensitivity (67.3%), whereas aspiration cytology had the lowest (51.9%). In the study by Grunwald et al. (20), the most specific methods were evaluated as aspiration cytology, biopsy, and galactography with a rate of 100%. MRI was reported to have the lowest specificity (25%) and the highest sensitivity (65.2%). Ductoscopy was evaluated as having 55.2% sensitivity and 61.5% specificity, along with US. Albrecht et al. (21) reported 60% sensitivity and 66.7% specificity for MRI, 53.2% sensitivity and 60% specificity for ductoscopy. Ohlinger et al. (22) results were 82.5% sensivity and 11.8% specificity for MRI, different from the literature; ductoscopy was reported as having 71.2% sensivity and 49.4% specificity. In our study, both magnetic resonance (90%) and ductoscopy (94.6%) in the diagnosis of ID lesions resulted in high sensitivity when compared with the literature (18,19,20). The specificity of MRI (66.7%) was found quite high compared with the literature and equal to the results of Albrecht et al. (21).

In our study, after considering that both dMRG and ductoscopy would be difficult to tolerate by patients because of their invasive nature, idMRG was used. We performed sagittal heavily T2-W fat-suppressed sequences to show ductal tree visualization; dilated ducts can be observed as high-signal tubular structures and ID lesions as filling defects. These images are similar to galactography. Also, 3D T1-W TFE sequence-dynamic images were obtained in sagittal planes and evaluated using T2-W images together. Contrast-enhanced lesion with ductal tracing on subtraction image and ID lesions as the filling defects on T2-W image were combined; this evaluation allowed diagnosis or strengthened our diagnosis.

In a study by Daniel et al. (25), MR images of solitary ID papilloma were described as: 1- small luminal mass papillomas; 2- tumour-like papillomas; and 3- MR-occult papillomas. Papillomas that do not enhance any contrast and that do not contour in T2-W images can be classified as MR-occult papillomas, which, in this study, explained our MRI-negative patients who were diagnosed as having ductoscopy and pathology. Several studies reported varying MR-occult papilloma rates; however, in a study by Son et al. (26), all disease was detected using MRI, and MRI sensivity was reported as 100% (26). Tominaga et al. (27) classified papillomas into four MRI types by grouping them according to their pathologic features; solid and cystic masses were reported in addition to the literature. In our study, ID papillomas were observed as oval nodules that showed well-circumscribed smooth margins within dilated ducts. All papillomas were located in the subareolar region and classified as central. These features of papillomas were reported especially in patients with symptoms of nipple discharge (27,28). It was reported in the literature that papillomas often rapidly enhanced contrast in the early phase, and that they reached a plateau or had delayed washout in dynamic analysis (14,15,25). In our study, however, persistent-type contrast enhancement was shown in all papillomas that were classified as central. The diversity of enhancement patterns in central and periphery papillomas may have caused the differences in the dynamic analysis results. Further research is needed to confirm this finding.

This study is limited because of the number of patients. It was relatively small; however, the participation of only one surgeon and only one radiologist in the study led to a more standardized and effective evalution.

In the light of developments in MRI technology and extensive studies on ID lesions, MRI plays a valuable complementary role in addition to standard diagnostic methods in researching the aetiology of PND. However, MRI should not be the only method in evaluating continuing PND because it is likely that there will also be MR-occult ID lesions; therefore, it should be used alongside ductoscopy. As reported in our study and also in the literature, visualizations of ID papillomas and papillomatosis vary; hence, it is not always possible to discriminate the benign from the malignant.

## CONCLUSION

MRI and ductoscopy were superior to US in the diagnosis of PND. In our study the superiority of MRI to ductoscopy in the diagnosis of ID lesions was not statistically significant. However, because MRI is a non-invasive method it is more advantageous for showing ductal tree visualization and is able to detect completely obstructed ID lesions. Breast MRI is a valuable diagnostic method with the finding of a sensitivity over 90% in the identification of ID lesions and further research about the aetiology of pathologic nipple discharge should be performed in this group of patients.

## Figures and Tables

**Table 1:**
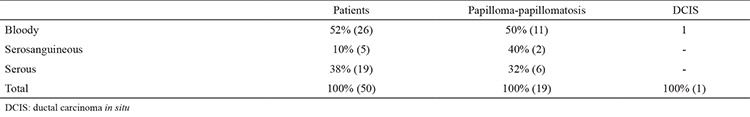
Frequency of types of nipple disharge (n=50)

**Table 2:**
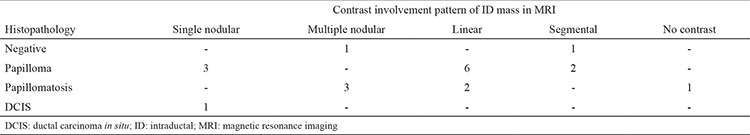
The comparison of MRI findings and histopathologic results in the diagnosis of intraductal mass (n=20)

**Table 3:**
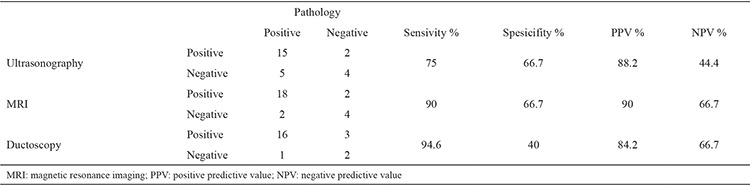
Comparison of results of ultrasonography, magnetic resonance imaging and ductoscopy with histopathology in the diagnosis of intraductal masses
